# A Comparative Study of Peri-Operative Fluid Therapy With Ringer Lactate and PlasmaLyte in Children Undergoing Intra-Abdominal Surgery: A Randomized Control Trial

**DOI:** 10.7759/cureus.39124

**Published:** 2023-05-17

**Authors:** Andal Priyanka, Usha Ganapathy, Rajneesh Choudhary, Santhosh Arulprakasam, Rakhi Krishna C M, Jeevasri Calairadjane

**Affiliations:** 1 Anesthesia and Critical Care, Vardhman Mahavir Medical College and Safdarjung Hospital, New Delhi, IND; 2 Anesthesia and Critical Care, Jawaharlal Institute of Postgraduate Medical Education & Research, Puducherry, IND

**Keywords:** fluid therapy, paediatric abdominal surgeries, perioperative, plasmalyte, ringer lactate

## Abstract

Background

In this study, we compared Ringer’s lactate solution (RL) with PlasmaLyte (PL), a relatively new IV fluid, for perioperative fluid therapy in the pediatric population.

Methods

This prospective and interventional randomized comparative study was carried out after obtaining clearance from the Institutional Ethics Committee. The study period was from November 2016 to December 2017.

Results

Hemodynamic parameters such as SpO2, ETCO2, heart rate, blood pressure, temperature, and urine output were stable in both groups throughout the perioperative period without any statistically or clinically significant variations. Children receiving PL (group PL) had better acid-base status, serum electrolytes, and blood lactate profiles compared with children receiving RL (group RL), who had hyponatremia and increased blood lactate levels, which continued to increase in the immediate postoperative period. No significant differences in pH, pCO_2_, HCO_3_, serum potassium, serum chloride, blood urea, serum creatinine, or blood sugar were observed.

Conclusions

PL is better than RL for perioperative fluid therapy in children undergoing abdominal surgeries.

## Introduction

Ringer’s lactate (RL), a BSS, is a commonly used IV fluid for fluid therapy in both adults and children. However, it is not exempt from aftereffects, such as hypotonicity, hyponatremia [1,2], and hyperlactatemia [3-7]. Because of its harmful effects on acid-base abnormalities, electrolytes, and plasma tonicity, a new balanced salt solution, PlasmaLyte (PL), was developed. In this salt solution, the lactate buffer (which is found in Ringer's lactate) was replaced with acetate and gluconate as buffers for getting converted into water, carbon dioxide, and bicarbonate. PL is similar to plasma in terms of pH, osmolality, and electrolyte composition [8]. The choice of using PL includes the correction of electrolyte and volume deficiencies while dealing with acidosis and lactate accumulation [9].

Because of the differences in body composition and functions of pediatric patients, their fluid management differs from that of adults. This difference makes managing fluid therapy for pediatric patients an intricate task [10]. Pediatric patients undergoing abdominal surgery comprise a large number of in-hospital admissions each year [11]. The inimical effects of prolonged fasting, bowel preparation, pathological fluid sequestration, prolonged bowel exposure, and blood loss make children undergoing abdominal surgeries susceptible to hemodynamic instability, dyselectrolytemia, and acid-base disturbances [12]. The skepticism surrounding the knowledge about which fluid to use for pediatric patients and how much of it to administer perplexes caregivers. However, 0.9% and 0.45% saline solutions are routinely used in the pediatric age group both for maintenance and replacement therapy. Controversies persist regarding the ideal type, dose, and timing of IV fluid administration in perioperative fluid therapy in children because well-controlled studies comparing various IV fluids for perioperative fluid therapy in pediatric patients are scarce. Hence, we conducted a study comparing the routinely used RL solution with PL, a relatively new IV fluid, for perioperative fluid therapy in the pediatric population.

## Materials and methods

This prospective and interventional randomized comparative study was carried out after obtaining clearance from the Institutional Ethics Committee. The period of study was from November 2016 to December 2017. The procedures followed were in accordance with the ethical standards of the Helsinki Declaration.

In the absence of a previous study on pediatric perioperative fluid therapy, the sample size of two samples with a continuous outcome variable was calculated using Cohen’s effect size. To detect medium-scale ES (.65), with 80% power of the study and a two-sided alpha of 5%, the minimum required sample size was 38 patients per group; hence, the sample size was 80 participants (40 per group) [13]. Children of either sex undergoing abdominal surgery were randomly allocated into one of the two groups (n = 80) using computerized block randomization.

The participants were randomly divided into two groups. In total, 80 children were screened, completed the study, and followed up. Group RL (n = 40) was administered RL as intravenous fluid. Group PL (n = 40) was administered PL as intravenous fluid. We included American Society of Anesthesiology (ASA) grade 1 children, children between 5 and 12 years of age, children of either sex and children undergoing elective intra-abdominal surgery. We excluded children with severe dehydration and electrolyte imbalance; children with hemodynamic instability, coagulopathy, or any hematological diseases; children undergoing surgery for less than one hour; children with acute or chronic renal/hepatic disease; children with preoperative bowel preparation; children with mitochondrial diseases; children having diuretics, acute gastroenteritis, or fever; children with seizure disorders; and children with hypersensitivity to intravenous fluids.

Randomization

**Figure 1:**
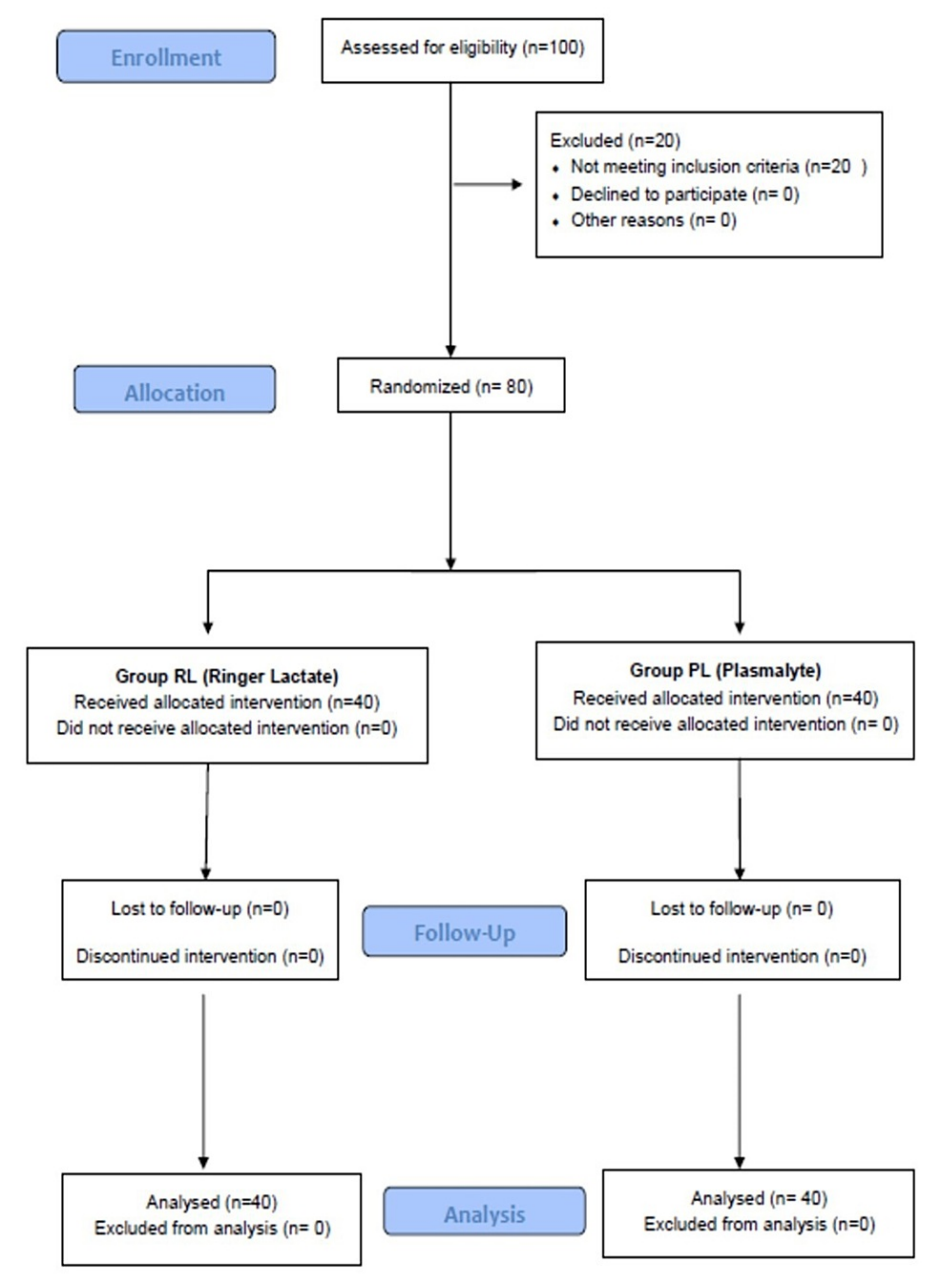
CONSORT diagram of the study

Computerized block randomization was done. We considered blocks of size four-that is, for every four patients randomized, two received perioperative fluid therapy with ringer lactate, and the other two received PlasmaLyte. There were six different ways that four patients were split evenly between two treatments: 1) AABB, 2) ABAB, 3) ABBA, 4) BAAB, 5) BABA, and 6) BBAA. Now to randomly select among these six different blocks, the random number generating function RAND Between was used with a lower limit of 1 and an upper limit of 6. Blocks were generated according to the number generated, if 5 was generated, then the BABA sequence was used, and so on. Allocation concealment was done using a sequentially numbered opaque sealed envelope (SNOSE). The principal investigator generated a random number, assessed it for eligibility, and assigned it to either of the two groups (allocation ratio 1:1) but was not involved in data collection. The RL and PlasmaLyte were de-identified and given several registrations (A.B.). The anesthesiologist conducting the study and the patient receiving the fluids were blinded. Figure 1 shows the CONSORT diagram of the study.

Preoperative evaluation and premedication

All the children underwent a detailed pre-anesthetic evaluation at the PAC clinic. A detailed history of their present, their past, and any medication use was taken, and a thorough physical examination was performed. The demographic and anthropometric parameters were measured and recorded. A detailed assessment of the airway was performed to rule out airway difficulties. All the children underwent routine blood examinations, including a complete hemogram, random blood sugar, acid-base status, serum electrolytes, renal function test, bleeding time, and clotting time.

The nature of the trial was explained to the participants in their native language, and written informed consent was obtained from their parents or responsible attendants willing to participate in the study. All the children were allowed to consume solid foods orally until six hours before anesthesia and clear fluids until two hours before the scheduled time for anesthesia. Syrup predictory (triclofos sodium) was given to all the patients as premedication. Children between 1 and 5 years of age were given 0.25-0.5 gm, and those between 6 and 12 years of age were given 0.5-1 gm.

Statistical analysis

Categorical variables are presented as numbers and percentages (%), and continuous variables are presented as mean ± SD and median. The Kolmogorov-Smirnov test was used to test the normality of the data. Non-parametric tests were used if normality was rejected. Quantitative variables were compared using the unpaired t-test/Mann-Whitney U test for non-normal distribution between the two groups and the ANOVA/Kruskal-Wallis test for non-parametric data. Qualitative variables were compared using the chi-square test or Fisher’s exact test. A value of P less than 0.05 was considered statistically significant. The data were entered in a Microsoft Excel spreadsheet (Redmond, USA), and the analysis was performed using IBM Corp. Released 2012. IBM SPSS Statistics for Windows, Version 21.0. Armonk, NY: IBM Corp.

## Results

The mean age of the children in the RL group was 8.1 years, and that in the PL group was 7.9 years. Our study included 44 male children and 36 female children. The sex of the children was comparable between the two groups and was not statistically significant (P = 0.839). The mean weight of the children in the RL and PL groups was 25.4 kg and 25 kg, respectively, and was comparable and statistically significant (P = 0.748). The mean height of the children in the RL group was 129.9 cm, and that in the PL group was 121.7 cm; it was not statistically significant (P = 0.795). No significance was found when the mean duration of surgery in the RL and PL groups were compared (P = 0.644), which were 182 minutes and 170 minutes, respectively. Most children in both groups underwent large bowel surgery, and this was not statistically significant (P = 0.461).

The trend of blood urea between the groups

No significance was found in the blood urea levels (mg/dl) between both groups throughout the study (P > 0.05).

The trend of serum creatinine between the groups

No significance was noted in serum creatinine levels (mg/dl) between the two groups (P > 0.05).

The trend of serum sodium between the groups

**Figure 2:**
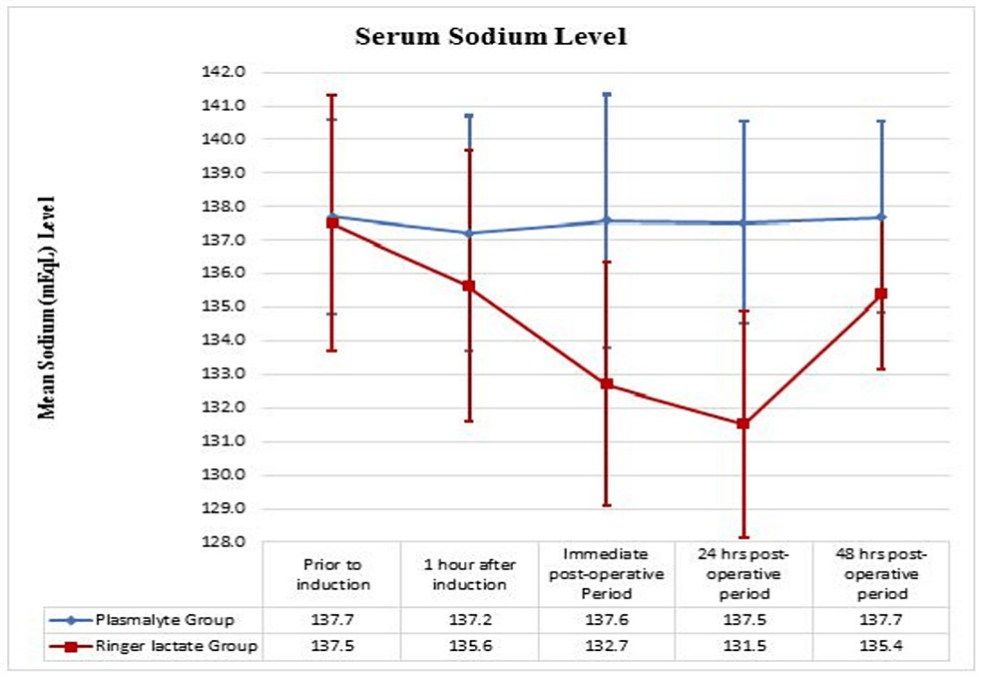
The trend of serum sodium between Ringer Lactate and PlasmaLyte group

Serum sodium levels (mEq/L) were comparable between both groups and were not statistically significant before induction. The serum sodium level revealed a progressive decrease in the RL group in the postoperative period, and a significant mean difference was observed in the serum sodium level in the immediate postoperative period (P = 0.00), 24-hour postoperative period (P = 0.00), and 48-hour postoperative period (P = 0.00) (Figure 2).

The trend of serum potassium between the groups

**Figure 3:**
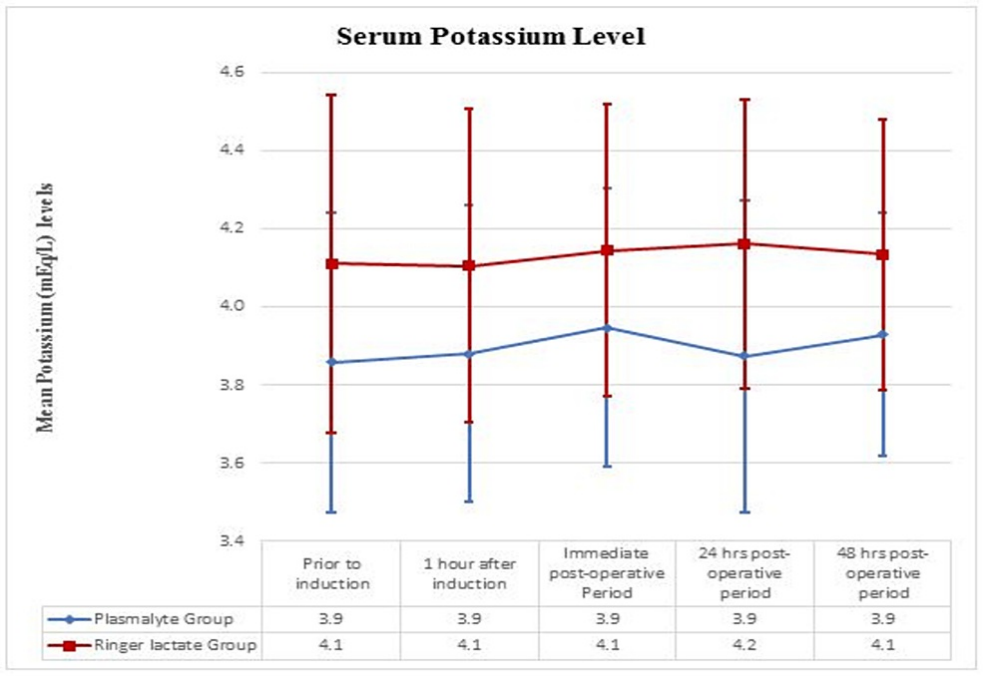
The trend of serum potassium between Ringer Lactate and PlasmaLyte group

A significant difference was noted in the serum potassium levels (mEq/L) between the two groups (P < 0.05) but within the normal limit (Figure 3).

The trend of serum chloride between the groups

No significant difference was seen in the serum chloride levels (mEq/L), and they were comparable between the groups throughout the study (P > 0.05).

The trend of blood sugar between the groups

No significant difference was noted in the blood sugar levels (mg/dl) between the two groups throughout the study (P > 0.05).

The trend of pH between the groups

No significant difference was noted in the pH values between the two groups throughout the study (P > 0.05).

The trend of pCO2 between the groups

**Figure 4:**
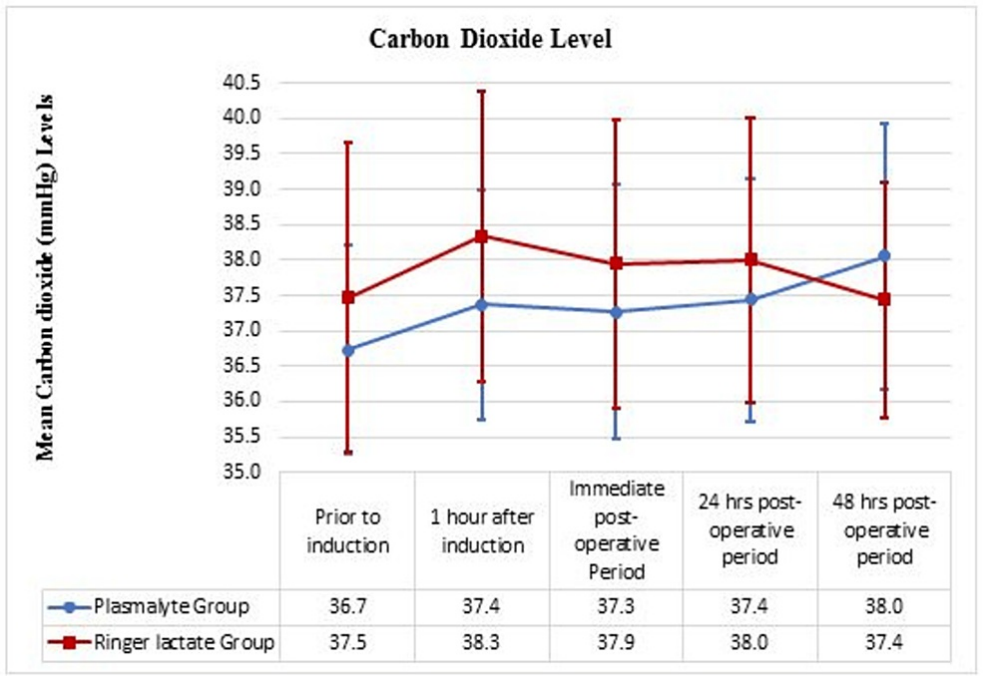
The trend of pCO2 between Ringer Lactate and PlasmaLyte group

Before induction, in the immediate postoperative period, the 24-hour postoperative period, and the 48-hour postoperative period, no significant difference was noted in pCO2 levels (mmHg) between both groups. There was an increase in pCO2 levels one hour after induction, and the difference in the mean level of pCO2 was significant (Figure 4). 

The trend of HCO3 between the groups

Serum bicarbonate (mEq/L) was comparable between the two groups throughout the study and was not significant (P > 0.05).

The trend of blood lactate between the groups

**Figure 5:**
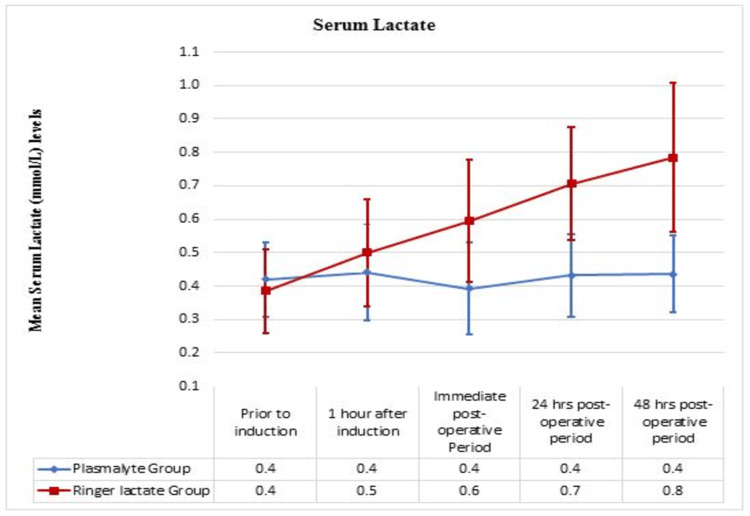
The trend of serum lactate between Ringer Lactate and PlasmaLyte group

Blood lactate levels were comparable between the two groups before induction and one hour after induction and were not significant. A significant and progressive increase in blood lactate levels was seen in the RL group, and the difference in the mean level of blood lactate was significant in the immediate postoperative period (P = 0.01), the 24-hour postoperative period (P = 0.00), and after 48 hours of the postoperative period (P =0.00) (Figure 5).

## Discussion

In a multicenter, prospective, double-blind, randomized controlled trial by Weinberg et al. (2015) that compared the acid-base status, biochemical, and hematological effects of PL and RL in 60 patients undergoing liver resection, an increase in blood lactate levels (P = 0.02) and hyperchloremia (P = 0.01) were observed in patients receiving RL. There was no discrepancy in the base-excess values. A decrease was observed in the duration of in-hospital stays and the rate of complications in the PL group. Our study also showed that patients receiving RL had increased lactate levels in the immediate postoperative period (P = 0.01) that tended to increase further in the 24-hour period (P = 0.00) and the 48-hour postoperative period (P = 0.00) [14].

A randomized controlled study conducted by Shin et al. (2011) comparing perioperative fluid therapy with RL and PL in 104 adult patients undergoing hepatectomy for organ transplantation revealed that serum lactate levels were higher in the RL group (P = 0.005) than in the PL group. Our study also revealed similar findings of increased lactate levels during fluid therapy in the RL group. However, Shin et al. studied young, healthy adults undergoing the right hepatectomy for living organ donations, whereas our study included ASA grade I pediatric patients aged 5-12 years who underwent elective intra-abdominal surgery [15].

Liu Xiaoyan et al. (2009) compared the effects of crystalloids on glycometabolism in 40 adult patients with type 2 diabetes mellitus undergoing gastrointestinal surgery, divided into two groups: RL (n = 20) and PL (n = 20). They observed hyperlactatemia in the patients receiving RL. At the end of the surgery, they found no change in HCO3, pH, base excess, or pCO2 in either group. They suggested that PL is the safest and most efficient crystalloid solution for patients with diabetes during surgery. The outcome of our study was similar to that of theirs; however, our study population included children undergoing abdominal surgery [6].

Another study comparing PL, RL, and NS 0.9% by Hadimioglu et al. (2008) in 90 adult patients undergoing renal transplantation showed that the NS group had hyperchloremic metabolic acidosis and that the patients receiving RL had a progressive increase in blood lactate levels (P-value not stated). They concluded that PL had a better metabolic profile than RL or NS in patients undergoing renal transplantation. Our study also showed similar results in terms of the increase in blood lactate levels in the patients receiving RL. Unlike their study, we did not compare NS with other fluids in pediatric patients undergoing elective intra-abdominal surgery [16].

In a randomized controlled study by Takil et al. (2002), comparing NS and RL in 30 adult patients undergoing spine operation, NS was related to hyperchloremic metabolic acidosis, and RL was related to a decrease in serum sodium concentrations with hypercapnia. The mechanism was explained by the dilution of sodium with massive RL infusion and lactate metabolism to HCO3 and CO2, which led to hypercapnia and a drop in pH. The results observed in their study were similar to those of our study in terms of hyponatremia in the patients receiving RL. Our study also showed that the decrease in serum sodium was statistically significant in the immediate postoperative period (P = 0.00), 24-hour postoperative period (P = 0.00), and 48-hour postoperative period (P = 0.00) in the patients receiving RL. The phenomenon of decreased serum sodium could be attributed to the hypotonicity of the RL solution compared to that of PL, with subsequent free water retention [2].

A study by Scheingraber et al. (1999) comparing NS and RL in 24 adult patients undergoing lower gynecological surgery reported lower blood lactate levels and pH values in patients who received NS than those who received RL (P = 0.01). The secondary outcome of the study was that the patients who had received RL had decreased sodium and chloride levels in the postoperative period. The outcomes of their study were also confirmed in our study, namely, an increase in blood lactate and hyponatremia with RL administration. Their study differed from ours in that they compared RL and NS in adult patients undergoing gynecological surgery, while our study compared PL and RL in pediatric patients who had been planned for intra-abdominal surgery [1].

Chatrath et al. (2016) compared PL and NS for fluid-deficit replacement in intra-abdominal surgeries intraoperatively and their effect on arterial pH, HCO3-, serum electrolytes, 24-hour urine output, and hospital stay post-surgery. They randomly allocated 60 patients into two groups. The participants were administered either intravenous PL or NS during the surgery at 15 ml/kg/hr. Perioperatively and 24 hours after surgery, the blood gas and electrolyte levels were measured. A fall in pH (P < 0.001), an increase in chloride levels (P <0. 001), and an increase in sodium levels (P = 0.001) were noted in the NS group during the immediate postoperative period. They concluded that PL maintained a better acid-base and electrolyte profile in the immediate postoperative period. We also observed a better biochemical and acid-base profile with PL in the perioperative period, but our study compared PL with RL, not NS [17].

Limitations of the study

One of the limitations of our study is that it did not account for the fluid intake in the perioperative period, which would have given a more comprehensive explanation for the outcomes, especially in the case of abdominal surgeries, and further studies are needed to analyze this in the future.

## Conclusions

The patients who had received PL had a similar acid-base status to those who had received RL but developed less hyperlactatemia and hyponatremia. RL is hypo-osmolar to plasma; in contrast, PL is a balanced salt solution, which has electrolyte contents comparable with those of plasma and is not associated with a disturbance of the acid-base status. Our study adds to the growing body of evidence suggesting that PL is more physiologically compatible with human plasma than RL and is associated with better outcomes. Hence, we conclude that PL is the preferred fluid for perioperative fluid therapy in children undergoing abdominal surgery.
